# Neuromorphic algorithms for brain implants: a review

**DOI:** 10.3389/fnins.2025.1570104

**Published:** 2025-04-11

**Authors:** Wiktoria Agata Pawlak, Newton Howard

**Affiliations:** ni2o, Washington, DC, United States

**Keywords:** neuromorphic computing, brain implants, spiking neural networks (SNNs), mixed-signal design, neurocomputational models, brain-computer interfaces (BCIs), biohybrid interfaces, data compression

## Abstract

Neuromorphic computing technologies are about to change modern computing, yet most work thus far has emphasized hardware development. This review focuses on the latest progress in algorithmic advances specifically for potential use in brain implants. We discuss current algorithms and emerging neurocomputational models that, when implemented on neuromorphic hardware, could match or surpass traditional methods in efficiency. Our aim is to inspire the creation and deployment of models that not only enhance computational performance for implants but also serve broader fields like medical diagnostics and robotics inspiring next generations of neural implants.

## 1 Introduction

Neuromorphic computing is an interdisciplinary area that takes inspiration from biological neural systems to develop computing architectures and hardware. It emphasizes massive parallelism, energy efficiency, adaptability, and co-located memory and processing—contrasting with traditional von Neumann designs (Kudithipudi et al., 2025; Schuman et al., 2017). Neuromorphic Algorithms often incorporate phenomena like neuron spiking, synaptic plasticity, and network-level dynamics, aiming to replicate the brain’s style of processing. While some run on standard CPUs or GPUs, specialized neuromorphic chips—analog, digital, or mixed-signal—offer advantages for pattern recognition, sensory data analysis, and real-time learning by minimizing data-transfer bottlenecks (Kudithipudi et al., 2025; Schuman et al., 2022).

Implementing neuromorphic algorithms in hardware involves several steps:

1. *Algorithm design*: Formulating the mathematical models (e.g., spiking neurons, plasticity rules).
2. *Hardware architecture*: Selecting how neurons and synapses are represented, such as using analog or digital circuits
3. *Hardware description*: Employing design languages (e.g., Verilog) to produce implementable circuit specifications, though analog approaches may demand other tools.
4. *Chip fabrication*: Physically manufacturing the design, often in CMOS or emerging technologies like memristors.

Brain implants, meanwhile, are medical devices that interface directly with the brain’s bioelectrical environment. Currently, they are used for treating neurological disorders (Benabid, 2003), sensory prosthetics (Wilson and Dorman, 2008), motor prosthetics (Gupta et al., 2023), or mental health treatment (Reardon, 2017). Emerging and future applications include cognitive enhancement, brain-to-brain communication, neural rehabilitation such as rewiring neural pathways to restore function after stroke or brain injury (Baker et al., 2023; Chiappalone et al., 2022; Donati and Valle, 2024; Rao et al., 2014; Vakilipour and Fekrvand, 2024).

Integrating neuromorphic computing with these implants could enable more adaptive, low-latency control of neural signals. For example, a neuromorphic chip in an implant might adjust stimulation patterns in real time as brain states fluctuate, minimizing power consumption and improving clinical outcomes (Chiappalone et al., 2022; Donati and Valle, 2024). In this paper, we review neuromorphic models as well as neurocomputational models—ranging from single-neuron abstractions to large-scale network simulations—that appear well-suited for neuromorphic hardware. We also discuss how these models might address the unique demands of brain implants, such as biocompatibility, power constraints, and real-time adaptability in a living neural environment (Miziev et al., 2024).

## 2 The foundations and evolution of neuromorphic computing

From its beginnings, neuromorphic computing has sought to replicate the brain’s approach to handling information. In 1949, Donald Hebb proposed synaptic plasticity as a mechanism for learning and memory (Hebb, 1988) laying a conceptual foundation for brain-inspired hardware. Carver Mead took these ideas forward in the late 1980s, pioneering analog very-large-scale integration (VLSI) chips such as artificial retinas and cochleas that circumvented the “von Neumann bottleneck” by combining memory and processing in one place as opposed to traditional separation of CPUs and memory units (Mead and Conway, 1978; Mead, 1989).

Although Mead was not alone in brain-inspired computing, his mixed-signal designs revolutionized the field by reducing latency and allowing data storage and processing to occur simultaneously across the network. Unlike conventional computing, where explicit instruction sequences guide operation, neuromorphic systems derive their “program” directly from the network’s structure (Mead and Conway, 1978; Mead and Ismail, 1989; Schuman et al., 2017). Over time, the term “neuromorphic computing” has broadened covering a wider range of software and hardware implementations—digital, analog, and mixed-signal implementations (Mehonic et al., 2024; Schuman et al., 2022). In the 1990s and 2000s, neuromorphic chip development accelerated, driven by the DARPA SyNAPSE program. Researchers explored architectures for specialized applications like pattern recognition and sensory processing, including the use of memristors for more efficient, brain-like computation (Hylton, 2007; Markram, 2012). Key developments included silicon neurons, synapse models, and large-scale systems—all embodying plasticity and learning principles.

βIn the 2010s, there was a significant focus on advancing both hardware and algorithms including IBM’s “spiking-neuron integrated circuit” TrueNorth (Merolla et al., 2014), Neurogrid (Benjamin et al., 2014), BrainScaleS (Schemmel et al., 2020), Loihi (Davies et al., 2018), or SpiNNaker (Furber et al., 2012). These platforms modeled networks of spiking neurons achieving low power consumption and real-time processing for sensory data. Parallelly, the progression toward fully digital, event-driven neuromorphic chips was advanced by SynSense’s SENECA, ReckOn, Speck, and Xylo, allowing edge-based processing for applications like object identification and sensory processing tasks (Christensen et al., 2022; Yao et al., 2024; Tang et al., 2023; Bos and Muir, 2022). Moreover, commercial neuromorphic solutions have since emerged from companies like BrainChip and Innatera, demonstrating the practical viability of low-power, on-device deployments.

Since 2020, new gradient-based training methods for spiking neural networks (Lee et al., 2016; Eshraghian et al., 2023) and real-time evolutionary optimization (Ahmadi et al., 2024) have opened the door to tasks once dominated by deep learning on GPUs. Moreover, optical “memristors” are being explored for high-bandwidth neuromorphic machine learning (Duan et al., 2024; Nirmal et al., 2024). This continuing evolution is thoroughly reviewed in recent roadmaps such as Christensen et al. (2022), showing an even clearer trajectory toward highly efficient, brain-inspired computing platforms—progress that is particularly advantageous for brain implants, which require ultra-low power consumption, minimal latency, and on-chip learning.

## 3 Brain implants

### 3.1 Overview

Brain implants are medical devices designed to interface with the brain’s bioelectrical environment by either reading neural signals to restore lost functions or modulating activity to bypass damaged pathways. Although neurons primarily communicate through electrochemical signals (action potentials and neurotransmitters), studies show that mechanical forces, glial cell interactions, and even quantum phenomena may also affect brain function (Allen and Barres, 2009; Franze, 2013; Hameroff and Penrose, 2013; Lambert et al., 2012). These insights might contribute to our understanding of the “brain code,” possibly allowing better control of the bioelectrical properties of all cells within the brain, affecting brain functions or even cellular regeneration (Boys et al., 2022; Park et al., 2025; Shim et al., 2024; Tanikawa et al., 2023; Zhao et al., 2024).

Such implants could address a variety of disruptions, including protein or ion channel dysfunction, myelin loss, mechanical trauma, and glial cell abnormalities (Roa et al., 2023; Wang et al., 2022). Examples include restoring motor control in Parkinson’s disease (where the loss of dopamine neurons impairs movement), reducing epileptic seizures through responsive neurostimulation (detecting and preventing abnormal firing), and using visual or auditory prosthetics to bypass damaged sensory pathways (Fernandez, 2018; Gupta et al., 2023; Hartshorn and Jobst, 2018). By decoding neural signals, these devices can translate an individual’s intentions into commands for controlling prosthetic limbs, restoring mobility and providing a sense of embodiment (Donati and Valle, 2024). Moreover, implants could address cognitive impairments, as shown by Schiff et al. (2023), who used thalamic deep brain stimulation in traumatic brain injury patients to improve executive functions. Das et al. (2024) showed attention mechanisms in non-human primates via LFP and spiking data, suggesting specific stimulation patterns might enhance attention regulation. Systematic reviews indicate noninvasive methods can alleviate ADHD symptoms (Yin et al., 2024), hinting at broader potential for brain implants. Because each condition has its own pathophysiological features, implants need to adapt dynamically to changing states to deliver more personalized and effective therapies. Consequently, neuromorphic algorithms and compatible hardware that model neuronal communication—and can record and stimulate neural activity—might be crucial for advancing these targeted solutions.

### 3.2 Brain implant workflow

Currently brain implants follow a multi-step workflow—surgical insertion into the target region, neural signal recording, on- or off-chip processing, and sometimes neuronal stimulation. By directly interfacing with the brain, they can record or modulate neural activity for therapeutic or rehabilitative purposes.

**Figure 1:**
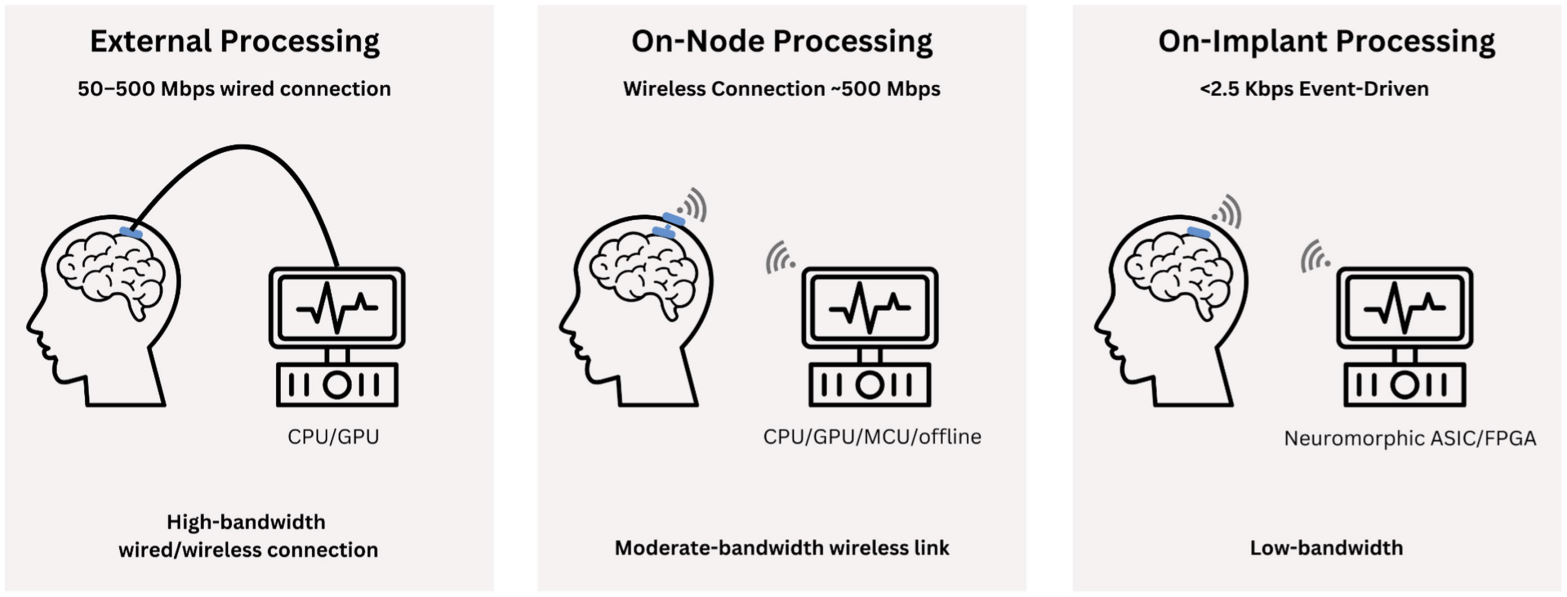
Illustrates the signal processing workflow for brain implants across the three tiers: external (on-workstation), on-node, and fully on-implant processing. Each tier highlights differences in hardware, bandwidth, tasks, and trade-offs between latency, power, and computational efficiency.

The typical workflow of a brain implant system involves:

1. Insertion of the implant: The device is surgically placed in the target brain area, with electrode placement varying based on the intended application. It is followed by a regeneration period since microglia are activated (Kozai et al., 2014).
2. Recording of neural signals: Electrodes on the implant detect the electrical activity of neurons. The resolution of this recording can vary significantly while recording:

a) Group of neurons (multi-unit activity)
  - Advantages: More stable signals over time; multi-unit recordings are often less susceptible to minor electrode shifts, which can occur due to micromovements of electrodes relative to the tissue (Ramezani et al., 2024; Supèr and Roelfsema, 2004).
  - Disadvantages: Less precise, may miss nuanced neural activity or possibility to directly modulate or bypass specific neurons (Gupta et al., 2020).
b) Single-neuron resolution (single-unit recordings)
  - Advantages: Highest precision, allowing for detailed neural decoding depending on the number of electrodes. This enables targeted stimulation and neuron-to-neuron algorithms for data processing and stimulation (Fu et al., 2016; Gupta et al., 2020; Zhang et al., 2023).
  - Disadvantages: More challenging to maintain long-term stability due to tissue response or electrode degradation (Kozai et al., 2014).

3. Processing of neural signals: The recorded data undergoes different signal processing steps to filter out noise and extract relevant features. This processing can occur in different locations:

a) On workstation processing:

- Recording: High-resolution electrodes capture neural signals.
- Processing: Data is sent to an external workstation for processing either with wire or wirelessly.
  - Advantages: High computational power. Large CPUs or GPUs can run advanced algorithms (e.g., deep learning or complex statistical methods), and workstation hardware and software are easier to update or replace (Zhang et al., 2022).
  - Disadvantages: Increased latency, reliance on external devices. While reliance itself is not inherently problematic (consider how our phones rely on satellites), it introduces challenges in data transmission, power transfer, and potential data bottlenecks (Ding, 2024). When processing occurs on an external workstation, data transmission can face challenges such as:
    - Signal attenuation and degradation over wireless transmission
    - Bandwidth limitations affecting real-time fast processing
    - Security and privacy concerns if sensitive neural data is sent to the cloud
    - Increased power consumption for data transmission
    - Potential loss of data during transmission
    - Reliance on continuous connectivity and compatibility with external systems, risking partial or total loss of functionality if communication is disrupted (Miziev et al., 2024).
  These factors can impact the system’s overall performance, reliability, and suitability for continuous, real-time neural signal processing in brain-computer interfaces (Lebedev and Nicolelis, 2006; Schalk et al., 2004).

b) On-node processing:

- Recording: Electrode arrays record neural activity.
- Processing: Local processing occurs on the node, with external data transfer for further analysis or on-station synchronization.
  - Advantages: Potential for reduced latency for real-time neural signal processing compared to workstation processing, though this depends on the specific chip used. Enhanced privacy, as much of the data can remain on the local device rather than being transmitted to the cloud improving reliability through reduced network dependence, which helps avoid data corruption associated with sending signals to and from external CPUs (Miziev et al., 2024).
  - Enhanced privacy by keeping data local, and improved reliability through reduced network dependence, which helps avoid data corruption (Miziev et al., 2024).
  - Disadvantages: Limited by node processing power. The lack of cloud connectivity means the AI might rely on a single individual’s data rather than aggregated data from many users. Additionally, the power consumption of on-board chips can limit computational speed, as higher clock speeds demand more energy and might lead to increased heat, potentially resulting in tissue damage if the temperature rises. Updating algorithms and software for implanted devices also poses challenges (Miziev et al., 2024; Serrano-Amenos et al., 2023).

c) On-implant processing:

- Recording: High-density electrodes integrated into the implant.
- Processing: All signal processing occurs on the implant with possibility for external data transfer if needed.
  - Advantages: Minimal latency, real-time processing capability, and reduced data transmission requirements (Ding, 2024; Miziev et al., 2024). Additionally, patient privacy can be further protected by restricting data flow to the implant itself.
  - Disadvantages: Severely limited by power and size constraints of the implant and potentially increased temperature in the brain environment. The implant could process approximately 2 GB of data locally, eliminating the need for time-consuming data transmission (*at 1,024 channels sampled at 20 kHz with 10-bit resolution, a minute of uncompressed data could reach about 2 GB). However, due to power restrictions and safety regulations, the on-board chip operates at a slower speed than external processors. While sending data externally would incur transmission delays, external devices can still process data much faster (Musk, 2019; Stanslaski et al., 2012; Figure 1).

4. Stimulation: Based on the processed information, implant may stimulate specific brain regions. The decision to stimulate and the parameters of stimulation (frequency, intensity, duration) can be determined either by on-implant algorithms or by external systems, depending on the implant’s design and capabilities.

### 3.3 Current limitations

#### 3.3.1 Algorithms and dynamic neural environments

Many brain implants use machine learning methods (e.g., deep neural networks, RNNs, SVMs) to interpret neural activity and enable communication with external devices (Chapin et al., 1999; Hochberg et al., 2012). Although these techniques have proved beneficial (e.g., in Neuralink prototypes such as the pig demo and monkey cursor control), they often rely on relatively simple models and fixed stimulation parameters that may not suit rapidly changing conditions of living neural tissue, its plasticity, or fluctuations in the brain’s state over a human lifetime (Gulino et al., 2019; He et al., 2020; Musk, 2019).

#### 3.3.2 Data rates, compression, and transmission

Another substantial issue is data compression. The amount of neural data processed by a brain implant can vary widely, depending on factors like the number of electrodes, the sampling rate, spatial and temporal resolution (fine-grained control for individual neurons), and whether the device monitors spike-level signals (sampled at 20–30 kHz to resolve individual action potentials) or local field potentials (LFPs). Issues such as data compression or wireless transfer can slow real-time feedback for rapid neural events (Gilja et al., 2012; Harrison, 2008; Liu et al., 2020; Prosky et al., 2021). At the lower end, a system with a handful of electrodes recording at a few kilohertz might handle kilobytes to megabytes of data per second, whereas a high-density array with hundreds or thousands of channels could generate tens to hundreds of megabytes per second.

For instance, Neuralink generates approximately 200 Mbps of electrode data from its high-density implant but can only transmit 1–2 Mbps via Bluetooth—meaning the implant operates at about 0.5% of its potential. Achieving the required compression ratio of over 200× and is often handled by feature extraction or on-chip compression—yet these techniques do not fully maximize the implant’s capabilities. Furthermore, these systems often struggle with power consumption, size constraints, and the need for external processing units, which can limit their practicality and scalability (Gulino et al., 2019; Miziev et al., 2024; Neuralink Compression Challenge, 2025).

#### 3.3.3 Materials and biocompatibility

Additionally, material constraints, thermal noise, and the possibility of requiring immune-suppressing therapies can affect data quality, device performance, and patient well-being (Miziev et al., 2024). Some wound-healing and material research suggests that specific stimulation patterns may enhance healing speed—possibly reducing implant rejection or tissue scarring—lowering the degradation of signal quality and improving the precision of neural recording, although these methods have not been extensively applied in clinical practice (Boys et al., 2022; Fani et al., 2023; Miziev et al., 2024). Polymer coatings, hydrogel encapsulation, and flexible bioelectronics aim to reduce foreign-body responses and provide stable, long-term performance for neuromorphic implants (Hwang et al., 2015; Polikov et al., 2005; Qi et al., 2023; Salatino et al., 2017).

#### 3.3.4 Scalability, complexity, adaptability

Most current implants remain relatively large, limiting improved spatial or temporal resolution (Miziev et al., 2024). The complexity of neural signals—firing patterns, synaptic plasticity, network-level feedback—requires precise placement and calibration to avoid undesired side effects, such as cognitive dysfunction or behavioral changes (Valle et al., 2024). Many devices do not self-adjust to evolving neural conditions without external control, reducing their capacity to stay aligned with the user’s needs in the long term, especially as the brain’s functional state changes due to learning, aging, or disease progression (Shanechi et al., 2016; Zhou et al., 2023).

Addressing these issues is important for advancing brain implants. Bio-inspired robotics, improved materials design, and neuromorphic computing—with event-driven, efficient on-chip processing—may pave the way to adaptive, biologically informed systems (Chiappalone et al., 2022; Qi et al., 2023; Zhang et al., 2024). Fully implantable devices could reduce latency and enhance real-time interactions, though obstacles such as energy management, heat dissipation, and data compression methods remain unsolved (Chiappalone et al., 2022; Zhang et al., 2022).

## 4 Neuromorphic computing for brain implants

### 4.1 Brain-inspired foundations of neuromorphic computing

The human brain is a computational marvel, reaching exaflop-scale performance while consuming just ~20 watts (Yao et al., 2024). This efficiency contrasts sharply with traditional computing architectures, which are approaching physical limits. Moore’s Law, predicting a doubling of transistors every 2 years, is slowing due to transistor miniaturization constraints (Schaller, 1997), and Dennard scaling, which maintained power density as transistors shrank, is also faltering, complicating efforts to boost performance without sacrificing energy efficiency (Horowitz, 2014). These trends highlight the brain’s computational edge and the need to draw inspiration from its design.

Key features of the brain’s paradigm include:

- *Massive parallelism*: Unlike the sequential processing of traditional computers, the brain handles distributed, simultaneous computations across billions of neurons (Schuman et al., 2017). While architectures like SIMD, MIMD, or Dataflow can excel at specific tasks (e.g., image processing, matrix operations), they still lack the versatility and energy efficiency of the brain’s parallel computation across a wide variety of tasks from sensory processing to abstract reasoning (Hennessy and Patterson, 2011; Roy et al., 2019).
- *Integrated memory and processing*: Traditional von Neumann architectures separate memory and processing, creating bottlenecks absent in the brain, where computation and memory are believed (until now) to be integrated at the synaptic level (Isik et al., 2024; Kastellakis et al., 2015).
- *Adaptability and learning:* The brain’s real-time adaptability and plasticity outstrip machine learning algorithms, which struggle with catastrophic forgetting and lack comparable energy efficiency despite for example incremental learning (Kirkpatrick et al., 2017; Aleixo et al., 2024; Sadegh-Zadeh et al., 2024).
- *Fault tolerance:* Biological networks remain functional despite significant neuron loss, as seen in Alzheimer’s patients retaining abilities with reduced brain mass (Su et al., 2016). Traditional systems, however, are vulnerable to single points of failure, though efforts using genetic algorithms aim to address this (Zlokapa et al., 2022; Su et al., 2016).
- *Handling noisy data*: The brain processes noisy, incomplete inputs effortlessly, using mechanisms like sensory substitution. In cases where one sensory modality is impaired, the brain can rewire itself to process information from other senses to compensate for the loss (Merabet and Pascual-Leone, 2009). While there have been advancements in machine learning, traditional systems still mostly require precise data (Caiafa et al., 2021).

Neuromorphic computing largely addresses these issues. While the Harvard architecture already separates data memory and program memory (Hennessy and Patterson, 2011), neuromorphic approaches offer support for dynamic and real-time data processing at high throughput and low energy consumption, avoiding continuous data transfers between discrete memory and processing units. This reduces the bandwidth limitations of current technologies. Compression methods in neuromorphic systems focus on essential spikes or relevant features, reducing data size and lessening the load on external devices or wireless links (Roy et al., 2019; Lee and Lee, 2020). Because information is handled locally, these pipelines can cut back on unnecessary transfers, enhance real-time compression, and improve overall performance (Schuman et al., 2017; Rhodes et al., 2019).

Neuromorphic systems can improve spatial and temporal resolution through bio-inspired architectures with high-density, low-power processing units, reflecting the brain’s ability to process information at multiple scales. This leads to improved spatial resolution by better mapping—fitting more sensors or compute elements into smaller areas—and boosts temporal resolution through parallel, event-driven operations, allowing real-time monitoring and minimal-latency stimulation of neural activity (Hall et al., 2012; Gonzalez et al., 2024; Peres and Rhodes, 2022). Such responsiveness is crucial for effective neuroprosthetic control or closed-loop interventions with quick feedback (Niu et al., 2020).

In a prosthetic limb, for instance, a neuromorphic processor can interpret signals from sensory receptors instantly and adjust motor commands in actuators, resulting in smooth, natural movements aligned with the body’s reflex responses—especially in dangerous situations where fight-or-flight responses matter (Niu et al., 2020; Song et al., 2024).

A key feature of these systems is event-driven computation, where processing occurs only in response to significant input changes or ‘events’, rather than continuous operation (Ji et al., 2023; Shahsavari et al., 2023). This approach has been deployed in vision-processing tasks on neuromorphic platforms like Speck where real-time, event-based sensing enables low-latency, energy-efficient object recognition (Yao et al., 2024). This approach is particularly effective in managing temporally sparse activity, which is useful in various applications, such as detecting rare events or monitoring long-term trends, ensuring that the system remains efficient and responsive only when necessary (Aboumerhi et al., 2023). This also could make the neuromorphic design more energy-efficient and improve power consumption, minimizing the thermal impact on surrounding brain tissues.

Incorporating synaptic plasticity mechanisms, particularly spike-timing-dependent plasticity (STDP), allows neuromorphic systems to learn and adapt from new stimuli (Bill and Legenstein, 2014). This learning capability, together with the systems’ inherent parallel processing, strengthens pattern recognition and sensory data handling—advantageous in plastic neuronal environments (Zhu et al., 2021; Büchel et al., 2022a,b). Recent research has extended on-device computing capabilities, achieving state-of-the-art performance in real-time audio tasks (e.g., speech recognition) and vision tasks (e.g., object detection), all with notable energy efficiency (Yao et al., 2024; Yik et al., 2025). The scalability of neuromorphic computing has been demonstrated in projects like SpiNNaker, which can simulate millions of neurons in real time and simultaneously across multiple chips (Furber et al., 2012; Davies et al., 2018). This scalability spans from small, energy-efficient sensors to comprehensive neural networks modeling complex behaviors. For example, DeWolf et al. (2016) employed spiking neural networks for robotic arm control, while others have explored neuromorphic sensory systems (Liu and Delbruck, 2010), speech recognition (Xiang et al., 2023), and energy-efficient image classification (Pawlak et al., 2024).

Additionally, neuromorphic systems often include elements of stochasticity, reflecting the probabilistic character of biological neural networks (Petrovici et al., 2016). This enhances robustness and adaptability to uncertain, variable environments. For example, a recent study on neuromorphic-based closed-loop neuroprostheses (Chiappalone et al., 2022) describes how real-time data processing, energy efficiency, and bio-inspired computation can help reestablish or substitute injured neural pathways, going beyond sensory or motor restoration and potentially enabling direct brain-level repair. The broad applicability of neuromorphic algorithms aligns with the type of information processing found in the brain. However, as the field advances, the focus is expanding beyond just hardware. There is a growing need to integrate neuromorphic systems with algorithms and real-world applications, increasing our understanding of neuronal communication models to allow for the development of even more advanced neural interfaces (Furber, 2016).

### 4.2 Mixed-signal design in neuromorphic systems

Mixed-signal design is one of the techniques improving the efficiency of neuromorphic computing, integrating both analog and digital circuitry to mimic the brain’s information processing. This approach combines the flexibility and precision of digital systems with the energy efficiency and continuous-time processing capabilities of analog circuits. Quan et al. (2023) demonstrated the effectiveness of mixed-signal neuromorphic circuits in implementing energy-efficient and space-efficient Spiking Neural Networks (SNNs) using 55 nm CMOS technology. The integration of analog and digital components allows neuromorphic systems to better model the brain’s parallel processing and adaptive learning capabilities while maintaining computational efficiency. For instance, Benjamin et al. (2014) developed Neurogrid, a mixed-analog-digital multichip system for large-scale neural simulations, showing the potential of this approach.

#### 4.2.1 Benefits of mixed-signal design

Analog circuits are highly energy-efficient, often performing specific computations with far lower power consumption than digital alternatives. Their continuous-time nature allows real-time processing of sensory inputs, which aligns with how biological systems operate (Indiveri et al., 2011). Additionally, the high density of neural elements in mixed-signal circuits supports more compact designs for neuromorphic systems (Moradi and Indiveri, 2013).

#### 4.2.2 Challenges of mixed-signal design

However, analog components are inherently more sensitive to noise and environmental variations, which can impact system reliability (Qiao et al., 2015; Schuman et al., 2017). Recent efforts have begun to address issues such as mixed-signal mismatch, particularly during training, through techniques like mismatch-aware training algorithms (Büchel et al., 2022a,b) and improved circuit design methodologies (Murray and Edwards, 1994). Addressing these issues is crucial for the future development of mixed-signal neuromorphic architectures (Roy et al., 2019).

### 4.3 Algorithm to hardware conversion trade-offs

Neuromorphic hardware can be categorized into analog, digital, or mixed-mode (analog/digital) systems. While analog designs offer benefits such as a smaller footprint and lower power requirements, digital approaches tend to be more adaptable and cost-effective, for example, for running large-scale SNN models (Indiveri et al., 2011; Seo and Seok, 2015). For instance, TrueNorth (Merolla et al., 2014) and Loihi (Davies et al., 2018) each exemplify large-scale digital neuromorphic chips, achieving energy efficiency and event-driven spiking at scale. Small-scale digital neuromorphic processors have also gained attention for their potential in edge computing applications, offering low-power, real-time processing capabilities (Yik et al., 2025). In addition, mixed-signal designs combine analog front-ends with digital back-ends, supporting continuous-time processing (Roy et al., 2019).

The traditional approach of using high-level programming languages like Python for neuromorphic algorithm development, followed by conversion to hardware, still comes with its challenges. Schuman et al. (2022) emphasize the importance of co-designing algorithms and hardware to fully benefit from the characteristics of neuromorphic systems. Direct implementation of neuromorphic algorithms on specialized hardware, rather than relying on software intermediaries, can lead to substantial improvements in energy efficiency and processing speed. This is particularly relevant in neurotechnology applications, where real-time processing of neural signals is crucial (Furber et al., 2012). However, recent work with field-programmable gate arrays (FPGAs) has shown potential for neuromorphic solutions. A study by Zhang et al. (2019) reported speed-ups compared to CPU implementations and lower power consumption compared to GPU-based systems when SNNs were placed directly on FPGAs (Javanshir et al., 2022). Benchmarking efforts, such as the NeuroBench project (Yik et al., 2025) and edge audio evaluations (Bos and Muir, 2024), show the efficiency of small-scale digital neuromorphic processors in low-power, on-device sensory processing, including real-time audio (e.g., speech recognition) and vision (e.g., object detection) tasks. These findings show their potential for power-sensitive applications requiring minimal latency and real-time performance such as implants.

## 5 Neuromorphic algorithmic approaches for brain implants

Neuromorphic computing can address existing brain-implant limitations, offering an approach that improves energy efficiency, information transfer, and adaptive behavior—possibly including future memory storage. We reviewed a range of neuromorphic algorithms, from traditional ones and potential hybrid methods to neurocomputational models not yet implemented. We showed how to optimize them for the demanding requirements of brain implants: real-time processing, low power usage, and adaptive learning in complex, noisy environments.

### 5.1 Spiking neural network algorithms

#### 5.1.1 Fundamentals of SNNs

Spiking Neural Networks (SNNs) have significant relevance in the development and implementation of brain implants due to their ability to model natural neural processes, offering several advantages for brain-computer interfaces (BCIs), such as decoding neural signals, sensory substitution, or better personalization (Roy et al., 2019; Liao et al., 2024). The conceptual roots of SNN algorithms trace back to the mid-20th century, inspired by the work of neuroscientists like Alan Lloyd Hodgkin and Andrew Huxley (Hodgkin and Huxley, 1952). The formal introduction of SNNs as we know them today is often attributed to Wulfram Gerstner and his colleagues in the 1990s. Gerstner’s work on the Spike Response Model (SRM) in 1995 provided a framework for describing the behavior of spiking neurons mathematically (Gerstner, 1995).

However, the term “Spiking Neural Network” gained prominence in the late 1990s and early 2000s, with papers by Wolfgang Maass and William Bialek contributing significantly to the field (Maass, 1996; Bouvier et al., 2019). This coincided with advancements in VLSI technology that made it feasible to implement large-scale spiking networks in hardware (Indiveri et al., 2009; Indiveri and Liu, 2015). SNNs closely model the information processing mechanisms of biological neurons through several key principles:

1. *Membrane potential dynamics*: Each artificial neuron in an SNN maintains a membrane potential, which is a time-varying state variable (Izhikevich, 2003). The membrane potential changes in response to input spikes and decays over time when no input is received. In brain implants, this might allow for more natural interaction with surrounding biological neurons (Zhang et al., 2021).
2. *Threshold and spiking*: When the membrane potential exceeds a certain threshold, the neuron “fires” or emits a spike. After firing, the neuron enters a refractory period during which it is less likely or unable to fire again (Zhang et al., 2021; Guo et al., 2022). For brain implants, this thresholding mechanism provides a natural way to filter out noise and focus on significant neural events, improving signal quality and reducing power consumption (Shah et al., 2024).
3. *Temporal integration*: Neurons integrate incoming spikes over time, allowing them to process temporal patterns in the input data (Takaghaj and Sampson, 2024). This helps brain implants interpret and respond to complex neural signals more accurately. Additionally, temporal integration stores some representation of the signal in an analog form: the membrane voltage (and its fluctuations) encodes input amplitude and timing, functioning like parallel weighting coefficients in machine learning. This parallel processing capability is important for brain implants, allowing them to handle the massive parallelism of neural computations efficiently (Peres and Rhodes, 2022; Müller et al., 2023).

The dynamics of a spiking neuron can be described mathematically using differential equations. One popular model is the Leaky Integrate-and-Fire (LIF) neuron, which could be used for efficient memory, as noted by Kim et al. (2023). The dynamics of the LIF neuron are governed by the following equation, which models the evolution of the membrane potential over time:

$$\tau \frac{dV}{dt}=-\left(V-V_{\mathrm{rest}}\right)+RI\left(t\right)$$

Where:

- (V): Membrane potential
- Vrest: Resting potential
- tau: Membrane time constant (tau = R C, where (C) is the membrane capacitance)
- (R): Membrane resistance
- (I(t)): Input current
- When (V) reaches the threshold Vth, a spike is emitted, and (V) is reset to Vreset.

In the context of brain implants, the LIF model provides a framework for simulating and interpreting neural signals. For example, the membrane time constant (tau) determines how quickly a neuron responds to stimuli, which can be tuned to match biological neural processing speeds, approximating different biological neurons. This is important for applications like prosthetics, where accurate and timely decoding of neural signals is essential for smooth motor control (Donati et al., 2019; Donati and Valle, 2024).

#### 5.1.2 Information encoding in SNNs

The concept of encoding information in spike timing and frequency, rather than continuous values, has its roots in the study of biological neurons. This shift in perspective arose as neuroscientists investigated how the brain represents and processes information. Work in this field includes Eric Kandel’s research in the 1960s on synaptic transmission and plasticity, David Marr’s theories on neural computation in the 1970s, and Moshe Abeles’ exploration of precise spike timing in the 1980s (Kandel and Spencer, 1968; Kandel, 1976; Marr, 1969). These foundational studies laid the groundwork for the encoding strategies now used in Spiking Neural Networks (SNNs).

In SNNs, information can be encoded in several ways, such as Time-to-First Spike (TTFS) coding, Phase coding, and Burst coding, as well as the following:

1. Rate coding: The average number of spikes over a time window represents the intensity of a signal. In brain implants, rate coding can be used to interpret sensory or motor signals (Liu and Delbruck, 2010; Guo et al., 2021). For example, the frequency of neural spikes detected in the motor cortex could be translated into the strength or speed of movement in a robotic limb (Gupta et al., 2023; Chapin, 2004).
2. Temporal coding: The exact timing of spikes carries information. Temporal coding is critical for real-time processing in brain implants, enabling precise interpretation of rapidly changing neural signals (Cariani, 2001). This could be especially useful for applications like auditory prosthetics, where timing plays a key role in speech recognition (Aldag and Nogueira, 2024; Saddler and McDermott, 2024).
3. Rank order coding: The order in which neurons in a layer fire encodes information. Rank order coding can improve efficiency in brain implants by prioritizing the most significant neural inputs, reducing computational overhead while maintaining accuracy. This approach is particularly beneficial in energy-constrained systems like neural prosthetics (Loiselle et al., 2006).

##### 5.1.2.1 Practical data-to-spike conversion

Rate/temporal/rank order describe intrinsic coding strategies in SNNs (or biological systems), while binning/spike-count/charge-injection are applied methods for converting external signals to spikes. This aspect of input encoding is often overlooked: translating numerical data (e.g., raw sensor signals) into spikes so that SNNs can process them. Recent work compares binning, spike-count encoding, charge-injection, and more complex hierarchical strategies, demonstrating that the best input encoding depends heavily on the task and hardware (Schuman et al., 2019). For brain-implant scenarios, choosing an appropriate encoding method can significantly impact power usage, latency, and overall decoding accuracy—potentially just as critical as the learning algorithms themselves.

##### 5.1.2.2 ANN-to-SNN conversion

It offers a complementary approach for creating spiking networks. Instead of relying on spike-based encoding from the outset, this technique converts pre-trained artificial neural networks (ANNs) into spiking equivalents—preserving learned weights and architecture (Wang et al., 2023). Such conversion could potentially allow the use of existing, highly effective deep learning models in brain implants while benefiting from the energy efficiency of SNN implementations (Yamazaki et al., 2022). For instance, an ANN trained for speech recognition could be converted to an SNN, potentially enabling low-power, real-time decoding of auditory signals in a cochlear implant. Researchers have demonstrated promising ANN-to-SNN conversions for image classification (Rueckauer et al., 2017), which could be adapted for neural image input in visual implants, though this specific application has yet to be tested.

#### 5.1.3 Learning and optimization algorithms

Developing efficient learning and optimization algorithms is critical for enabling neuromorphic computing in brain implant chips, where on-chip computing must balance power efficiency, real-time processing, and adaptability.

##### 5.1.3.1 Spike-timing-dependent plasticity

STDP is often regarded as an important learning rule in Spiking Neural Networks (SNNs), particularly for brain implants seeking a biologically plausible way to adapt (Indiveri and Liu, 2015; Lan et al., 2021). By adjusting synaptic strengths based on the timing of pre- and postsynaptic spikes, STDP allows implants to modify responses to a user’s neural signals over time.

$$\Delta W=\left\{\begin{array}{cc} A_{+}\exp\left(-\frac{\Delta t}{\tau_{+}}\right) & \mathrm{if}\;\Delta t>0\\ -A_{-}\exp\left(-\frac{\Delta t}{\tau_{-}}\right) & \mathrm{if}\;\Delta t<0 \end{array}\right.$$

where ΔW is the change in synaptic weight and Δt is the time difference between spikes. Parameters (A_+_, A_−_, τ_+_, τ_−_) shape the STDP curve and can be adjusted for the desired balance between strengthening or weakening connections based on the user’s neural activity.

Recent work by Subramoney et al. (2024) presents a new perspective on SNN learning: fast adaptation that does not rely entirely on synaptic plasticity. Instead, it draws on a combination of slower plastic changes and faster network dynamics, incorporating biologically inspired elements such as spike frequency adaptation (SFA)—observed in a significant portion of cortical neurons. This approach allows SNNs to learn in a single trial, aided by recurrent connections that support key network behaviors. Synaptic weights still represent broader information (such as priors or task structures), but the system’s adaptive properties stem more from real-time dynamics and SFA than from plasticity-based rules like STDP.

##### 5.1.3.2 SpikeProp

Introduced by Bohte in 2002, SpikeProp is a gradient-based learning algorithm for spiking networks that adjusts synaptic weights according to the timing of individual spikes. It could enable brain implants to learn complex mappings—such as translating neural signals into motor control for a prosthetic hand—using supervised learning approaches (Bohte et al., 2000). For on-chip computing, its timing-based update is essential for real-time processing of complex neural signals in tasks such as motor control or sensory processing (Shrestha and Song, 2014). Recent advancements have extended SpikeProp with event-based update algorithms that enable exact gradient computation, improving training accuracy and efficiency (Wunderlich and Pehle, 2021).

##### 5.1.3.3 ReSuMe (remote supervised method)

Introduced by Filip Ponulak in 2005, ReSuMe combines STDP with supervisory signals (Ponulak, 2005; Ponulak and Kasiński, 2009). In contrast to purely Hebbian or STDP-based approaches, ReSuMe adjusts synaptic weights to minimize the timing discrepancy between the network’s output spikes and the target output pattern. This makes it well-suited to scenarios where a reference or “correct” spiking pattern is available. ReSuMe could help implants learn from external feedback, such as from a physical therapist during rehabilitation, to improve neural signal decoding over time. As the user would practice a motor task (e.g., hand movements), the implant’s spiking network receives corrective signals that guide STDP adjustments, refining neural signal decoding over time continuously aligning the SNN’s spike outputs.

##### 5.1.3.4 Spike-based backpropagation and BPTT for SNNs

Recent work by researchers like Zenke and Ganguli (2018) applies backpropagation-like learning (through surrogate gradients) to spiking neural networks (SNNs), allowing them to learn complex tasks while preserving energy efficiency—essential for brain implants (Gygax and Zenke, 2024). This approach adapts traditional gradient-based methods to handle the discrete, event-driven nature of SNNs, enabling efficient training for applications like neural signal decoding and personalized therapies (Eshraghian et al., 2023; Lee et al., 2016). A closely related approach, Backpropagation Through Time (BPTT), extends these methods by modeling the temporal dynamics of SNNs, making it especially suitable for personalized therapies or rehabilitation (Bird and Polivoda, 2021; Nápoles et al., 2024). By adjusting synaptic weights based on user-specific neural activity patterns, BPTT could potentially optimize interventions such as deep brain stimulation for Parkinson’s disease, where precise timing matters for symptom management.

Additionally, other forms of plasticity and learning rules are being explored in SNNs. For example, some research focuses on unsupervised feature learning with winner-takes-all-based STDP (Ferré et al., 2018), while others investigate the dynamics of phase oscillator networks with synaptic weight and delay plasticity (Chauhan et al., 2022).

### 5.2 Advanced SNN algorithms and hardware implementation

Neuromorphic computing algorithms, particularly those based on Spiking Neural Networks (SNNs), show great promise for applications in brain implants. These algorithms model the brain’s natural processing mechanisms, offering potential improvements in efficiency, adaptability.

#### 5.2.1 Models

1. *Spiking convolutional neural networks (SCNNs)* adapt CNNs into a spiking format—typically via rate or temporal coding—for low power, real-time processing of visual or sensory inputs (Kheradpisheh et al., 2017). For example, SCNNs can transform retinal implant signals into spike patterns interpretable by the brain (Yu et al., 2020), forming a basis for edge vision–based visual prostheses (Yao et al., 2024). An unsupervised SCNN approach has also bridged the gap between artificial and biological neurons by extracting image features and using receptive field–based regression to predict fMRI responses (Wang et al., 2023). Another study shows SCNNs detecting anticipatory slow cortical potentials for braking intention via EEG, outperforming standard CNNs, EEGNet, and graph neural networks with over 99% accuracy (Lutes et al., 2024; Kheradpisheh et al., 2017). This suggests strong potential for real-time motor control in driver assistance or prosthetic applications.
2. *Spiking recurrent neural networks (SRNNs)* integrate spiking neurons with recurrent architectures to process temporal neural signals, maintaining an internal state for tasks like predicting speech or motor patterns (Bohnstingl et al., 2022; Yamazaki et al., 2022). They could enable closed-loop systems to anticipate epileptic seizures with targeted, energy-efficient stimulation, though clinical efficacy requires further study. Adaptive SRNNs, with multiple timescales and self-recurrent parameters, match or surpass classical RNNs in sequential tasks, offering sparse spiking and > 100x energy savings (Yamazaki et al., 2022), making them ideal for motor-control implants and real-time monitoring on neuromorphic hardware (Willsey et al., 2022; Samee et al., 2022).
3. *Spiking Feed-forward Neural Networks (SFNN)* trained with gradient-descent methods enable efficient pattern recognition and sensory processing while remaining compatible with low-power hardware (Bauer et al., 2023). Using surrogate gradients and temporal coding, these networks approximate continuous derivatives to backpropagate errors effectively, achieving high accuracy in tasks like visual classification with energy-efficient sparse spiking (Zenke and Ganguli, 2018). This makes them well-suited for brain implants decoding sensory inputs in real time (Contreras et al., 2023).

#### 5.2.2 Neuromorphic-specific technologies

Real-world deployment of brain implants demands low-power, adaptive, and reliable hardware. Technologies such as event-based processing, memristive learning, and non-volatile memory (NVM) offer essential solutions to these challenges.

##### 5.2.2.1 Event-based algorithms

Event-based algorithms process data only when specific events (e.g., spikes) occur, reducing power usage and extending device operational time. This approach is similar to the behavior of biological neurons, which fire only upon receiving significant inputs. For example, Posch et al. (2014) demonstrated a retinomorphic event-based vision sensor adaptable to various sensory modalities, including auditory signals, for energy-efficient, real-time implant applications. Event-driven spiking CNN hardware, such as the Speck platform, further enhances this capability by enabling low-latency, energy-efficient processing of sensory data, making it ideal for real-time brain implant applications (Yao et al., 2024).

##### 5.2.2.2 Memristive learning algorithms

Memristive learning algorithms use memristors—devices that retain their resistance state when powered off—to implement synaptic plasticity directly in hardware. This design enables on-the-fly learning with low latency and resembles the way biological synapses adjust their strength over time (Boybat et al., 2018; Huang et al., 2023). Multiple studies demonstrate the use of memristors to implement synaptic plasticity in hardware. For example, BiFeO₃ (BFO)-based memristive devices have been shown to support various long-term plastic functions, including spike timing-dependent plasticity (STDP), cycle number-dependent plasticity (CNDP), and spiking rate-dependent plasticity (SRDP) (Du et al., 2021). Moreover, the TS-PCM device demonstrates the ability to modulate its behavior based on stimulus history, similar to neuronal plasticity (Sung et al., 2022). These findings suggest the potential for devices that can adjust to a user’s neural patterns.

##### 5.2.2.3 Non-volatile memory technologies

NVM technologies provide efficient, persistent storage for synaptic weights. Three key NVM types with potential for brain implants are:

- *Phase-change memory (PCM):* Uses chalcogenide materials that switch between amorphous and crystalline states to store data. Its capacity to represent multiple resistance states allows for analog-like computation and persistent synaptic storage (Burr et al., 2016).
- *Resistive RAM (RRAM):* Also known as memristive memory, it changes resistance based on applied voltage, simulating synaptic plasticity. Its scalability and non-volatility suit high-density synaptic storage in space-constrained brain implants (Li et al., 2023; Wan et al., 2022).
- *Ferroelectric RAM (FeRAM):* Employs ferroelectric materials to store data without power, featuring fast read/write speeds and high endurance. However, integrating FeRAM into silicon remains a challenge as their chemical properties vary, potentially causing unwanted reactions (Mehonic et al., 2024).

These NVM technologies retain learned synaptic patterns without continuous power, reducing energy consumption and improving reliability for devices such as neural decoders in prosthetics or memory-enhancement implants However, their application in brain implants requires further research on biocompatibility, long-term stability in biological environments, and integration with neural tissue (Rathi et al., 2022).

### 5.3 Emerging and theoretical models

#### 5.3.1 Multimodal learning algorithms

Multimodal learning algorithms implemented in neuromorphic chips show great promise for brain-computer interfaces and intelligent robotics. They enable the simultaneous processing of various sensory inputs (e.g., visual, auditory, and tactile), modeling the human brain’s ability to integrate multiple sensory modalities (Krauhausen et al., 2024; Li et al., 2024a,b). Recent advancements include the development of artificial synapses capable of handling multiple stimuli, allowing parallel in-memory computing and low-energy AI processing (Li et al., 2024a,b). A bio-inspired approach using organic neuromorphic circuits has demonstrated real-time multimodal learning in robotic systems, enabling intelligent environmental interaction suggesting potential applications in sensory substitution for brain implants (Krauhausen et al., 2024). Despite difficulties of integrating multiple data streams within implant constraints, multimodal neuromorphic systems have potential to improve data comprehension, performance, and adaptability for sensory substitution or augmentation.

#### 5.3.2 Liquid state machines and echo state networks

LSMs and ESNs both belong to reservoir computing and can be adapted for spiking (neuromorphic) hardware. They enable energy-efficient, real-time processing of spatio-temporal data, useful for applications like seizure prediction or prosthetic control. Studies on SpiNNaker and Loihi-2 show high accuracy in visual classification tasks with low power usage (Patiño-Saucedo et al., 2022; Pawlak et al., 2024); for instance, one LSM reached 91.3% on CIFAR-10 at 213 μJ/frame (Pawlak et al., 2024). ESNs have also been implemented in memristor crossbar arrays, leveraging neuromorphic parallelism and efficiency (Hassan et al., 2017). Recent advances include modular ESNs for EEG-based emotion recognition, achieving improved performance without additional neural adaptation, suggesting potential for brain implants requiring real-time signal interpretation (Yang et al., 2024).

#### 5.3.3 Liquid neural networks

Liquid Neural Networks (LNNs), inspired by biological systems, incorporate differential equations into their activation functions to better describe neuronal membrane dynamics. Their adaptive design could make implants more versatile for changing brain needs, enabling continuous, label-free learning. LNNs have shown promise in robotics, autonomous vehicles, and healthcare, where Closed-Form Continuous-Time LNNs (CfCs) enable real-time analytics of complex patient data for earlier diagnoses (Nye, 2023).

## 6 Neurocomputational models of neuronal communication

Neurocomputational models describe computational principles and structures that model the human brain’s neural architecture. Although computational neuroscience traditionally emphasizes biologically plausible models and detailed physiology, it also inspires (and is inspired by) broader fields like connectionism, control theory, and machine learning (Davison and Appukuttan, 2022). For instance, convolutional neural networks (CNNs)—inspired by the visual cortex—have been adapted into spiking CNNs (SCNNs) for sensory prosthetics, including retinal and cochlear implants (Lindsay, 2020; Büchel et al., 2022a,b; Alsakkal and Wijekoon, 2025).

Neuromorphic computing shares the core goal of replicating the brain’s efficiency and adaptability. While medical applications (e.g., brain implants) could particularly benefit from this synergy due to requirements for low-power, real-time operation, neuromorphic systems also have broader uses in general AI and robotics (Donati and Valle, 2024; Schuman et al., 2022). There are various ways to categorize neurocomputational models—such as single-neuron modeling, neuron–glia interactions, and sensory processing (Linne, 2024; Jiang et al., 2024; Herz et al., 2006). However, in this chapter, we will follow the five-level framework proposed by Herz et al. (2006), as it clearly illustrates how models range from high-fidelity (detailed compartmental) to purely functional (black box), showing the trade-offs in complexity, efficiency, and applicability for potential use on neuromorphic chips for single-neuron dynamics.

### 6.1 Models

#### 6.1.1 Detailed compartmental models (level 1)

Detailed compartmental models subdivide a neuron into many sections (compartments) to represent how its spatial structure affects electrical and chemical activities (Koch, 1998). They typically rely on anatomical reconstructions, ensuring that features like dendritic branches, axons, and ion channel distributions are included in a realistic way. These approaches build upon Rall’s cable theory, which mathematically showed that voltage attenuation in dendrites spreads asymmetrically (Herz et al., 2006; Rall, 1977; Mainen and Sejnowski, 1996). By using numerical integration across many compartments, these models can reflect complex biophysical details, including active dendritic currents (e.g., calcium spikes) and backpropagation of action potentials (De Schutter and Bower, 1994; Roth and Bahl, 2009). However, modeling large dendritic trees may require over 1,000 compartments, leading to a very high-dimensional system of equations (Amsalem et al., 2020).

##### 6.1.1.1 Examples

- Multi-compartmental neuron models
  Used in tools like NEURON, emphasizing accurate geometry and ion channel placement (Friedrich et al., 2013).
- Cable Theory–Based Models
  Extend Rall’s equations to cover voltage and current flow along dendrites and axons.
- Thalamocortical neuron models
  Enhanced over time with additional ion channels (e.g., dendritic calcium currents) to study fast oscillations or pathological rhythms (e.g., in sleep disorders) (Wang et al., 2022; Destexhe et al., 1998).

##### 6.1.1.2 Key features

- Morphological details
  Incorporate anatomical reconstructions to see how shape and structure influence neuronal activity.
- High fidelity
  Reflect ion channel variability, dendritic integration, and axonal propagation with considerable detail.
- Numerical complexity
  Require solving large sets of differential equations, especially when dendritic trees are extensive (Ben-Shalom et al., 2021).
- Mechanistic insights
  Can produce testable ideas about how certain firing patterns or oscillations arise (e.g., Purkinje cell simulations suggesting an inhibitory current behind specific spike patterns) (Lumer, 1997; Santoro et al., 2024).

##### 6.1.1.3 Potential applications for brain implants

1. Precision in stimulation

By modeling how voltage spreads across dendrites and soma, these models could help predict where an implant’s electrical pulses might have the strongest effect (e.g., in Parkinson’s DBS). However, running such detailed computations in real time on an implant could be impractical due to the heavy processing load (Bingham et al., 2018; McIntyre and Foutz, 2013).

2. Understanding neural disorders

Disease-linked alterations (e.g., modified ion channel conductance in epileptic tissue) could be studied in a spatially precise manner, supporting customized stimulation strategies (Suffczynski et al., 2004). However, detailed pathological modeling still demands high-end computing resources, making on-chip simulations unlikely.

3. Predicting extracellular stimulation effects

Because these models show how electrical fields interact with the neuron’s shape, they could suggest how implants should deliver pulses for maximum benefit in treatments like DBS. The challenge might arise as calculating the effects across many neurons or an entire region can rapidly exceed computational limits (Hussain et al., 2024; Yousif and Liu, 2007).

##### 6.1.1.4 Constraints

These models often require high-performance computing or offline simulations, making them unsuitable for real-time neuromorphic hardware or large-scale networks (Amsalem et al., 2020). Moreover, including full dendritic and axonal detail for every neuron in a network is generally infeasible. Although detailed compartmental models do guide design and optimization, their detailed simulations continue to be impractical for on-chip use. As a result clinicians and engineers typically employ them offline to optimize factors such as electrode placement or stimulation patterns, then transfer simplified models or empirically derived parameters to the actual device.

#### 6.1.2 Reduced compartmental models (level 2)

Reduced compartmental models model the spatial details of neurons but still include key biophysical elements (e.g., voltage-dependent currents, somatodendritic interactions). They represent a compromise between the high detail of Level I (detailed compartmental) models and the computational simplicity of Level III (single-compartment) models. By keeping a limited number of compartments—often two or three—they provide more biological details than single-compartment approaches, yet they remain easier to analyze than fully detailed simulations (Herz et al., 2006; Izhikevich, 2006).

#### 6.1.3 Examples

- Leaky integrate-and-fire (LIF) neuron model
  Focuses on membrane leakage and spike generation with fewer parameters than more detailed models such as Hodgkin–Huxley (Hodgkin and Huxley, 1952).
- Izhikevich neuron model
  Uses a minimal set of equations to reproduce various firing patterns, making it more efficient than fully biophysical approaches.
- Two-compartment models (soma + dendrite)
  Divide a neuron into soma and dendrite (or further sections) to study phenomena such as homeostatic plasticity or binaural time difference detection in bipolar cells (Bush and Sejnowski, 1993).
- Simplified dendritic tree models
  Keep partial branch structures to model local dendritic processes without modeling every branch.

##### 6.1.3.1 Key features of this group

- Somatodendritic interactions
  Allow partial modeling of how dendrites and soma exchange signals, influencing bursts, spikes, or oscillations (Tomko et al., 2021; Bush and Sejnowski, 1993).
- Calcium dynamics (when included)
  Enable phenomena like stable firing rate switching or short-term memory without requiring an elaborate multi-compartment tree (Marcucci et al., 2018).
- Scalability and mathematical clarity
  Compared to Level I models, these designs often scale better for network studies (e.g., cortical gamma or slow-wave oscillations), and are simpler to analyze for emergent behaviors (Close et al., 2014).
- Task-specific computations
  These models can capture how neurons perform behaviorally relevant computations at multiple timescales, linking neural structure to function.

##### 6.1.3.2 Potential applications for brain implants

1. Sensory prostheses

Reduced complexity may support near real-time simulations of hearing or vision pathways, helping design implants that reflect some somatodendritic interactions. Yet they still demand more resources than single-compartment models, which can limit on-chip processing for very large sensory arrays.

2. Adaptive interfaces

By including calcium currents or partial dendritic structures, these models could adjust to patient-specific firing patterns or changes in neural state. However ongoing parameter tuning may require external computation, given implant hardware constraints.

3. Local circuit simulations

Modeling small or medium-sized networks could help predict how groups of neurons respond to stimulation, guiding more targeted interventions (e.g., in motor or cognitive prosthetics). While more feasible than Level I, simulating an entire cortical region in real time may still be beyond typical implant hardware capabilities.

##### 6.1.3.3 Constraints

Though they need fewer resources than fully detailed compartmental models, these approaches still use more computational power than single-compartment designs (Bush and Sejnowski, 1993). Large-scale, real-time simulations in implant devices may prove difficult under such demands. For mid-scale tasks, however, they could deliver sufficient neural detail for certain network studies or adaptive interfaces, without causing excessive computational load.

#### 6.1.4 Single compartmental models (level 3)

Single compartmental models represent each neuron as a point-like unit and do not include the spatial details of dendrites or axons. They focus on how ionic currents govern subthreshold behavior and spike generation in a consolidated way, making them computationally efficient for large-scale or real-time simulations. Despite omitting dendritic or axonal structure, they often provide a useful quantitative look at how key variables—such as membrane voltage and ion channel states—interact to produce neural firing patterns (Koch, 1998).

##### 6.1.4.1 Examples

- Hodgkin–Huxley model
  Considered the prototype for Level III. It concentrates on multiple ion channels in a single compartment, explaining subthreshold dynamics and spike initiation without spatial subdivisions (Hodgkin and Huxley, 1952).
- Simple integrate-and-fire (if) model
  Focuses on input integration and threshold-based spiking, with minimal parameters.
- Leaky integrate-and-fire (lif) model
  Adds a leak term to better reflect real neural membranes.
- Theta neuron model
  Uses phase variables to track spiking behavior under minimal assumptions.
- FitzHugh–Nagumo model
  Includes simplified equations that approximate the action potential mechanism, often for conceptual or educational use.
- Izhikevich model (single-compartment form)
  Although often considered in the “reduced compartmental” category, it can also be implemented in a single-compartment form for certain use cases giving a more realistic representation of passive membrane properties (Izhikevich, 2003).

##### 6.1.4.2 Key features

- Removing spatial structure
  All dendrites and axons are lumped into one computational node, focusing on how combined ionic currents drive spikes. This distinguishes these models from Level I and II, which include some morphological detail.
- Quantitative understanding of dynamics
  They clarify how membrane voltage, ion channels, and thresholds govern phasic spiking, bursting, or spike-frequency adaptation—often through phase-plane or bifurcation analysis (Rinzel and Ermentrout, 1989).
- Mathematical reductions
  Systematic methods reduce or approximate more detailed models (like Hodgkin–Huxley) to an Integrate-and-Fire or resonate-and-fire form, enabling analytical insight (Izhikevich, 2006).
- Stochastic dynamics and noise
  Ion channel noise or background synaptic inputs can be included, explaining variations in spike timing and how random fluctuations might affect signal reliability.

##### 6.1.4.3 Potential applications for brain implants

###### 6.1.4.3.1 Basic neural communication

Because these models avoid spatial complexity, they could process large numbers of neurons with minimal computational cost, fitting power-limited implant constraints (Dehghanzadeh et al., 2021). However the absence of dendritic or axonal structure means these models might not reflect certain detailed processes relevant to specific therapies.

###### 6.1.4.3.2 Motor control implants

A lightweight design could be useful for decoding or controlling muscle activation patterns, potentially helping with real-time prosthetic limb control. Nevertheless adaptation or conduction delays that depend on neuron geometry are not represented, so precision in controlling multi-joint movements may be affected.

###### 6.1.4.3.3 Large-scale population simulations

Integrating thousands of these neurons for cortical assemblies or multi-region models is more feasible than with Levels I or II, which could be useful for broad network simulations within implant hardware. However, missing spatial interactions can reduce fidelity when studying phenomena that hinge on local dendritic integration or traveling waves.

###### 6.1.4.3.4 Rapid network responses

Low overhead could support fast feedback loops for closed-loop seizure detection or adaptive deep brain stimulation. However, oversimplified stochastic elements and nonlinearities may affect accuracy in complex pathological conditions.

##### 6.1.4.4 Limitations

Though single-compartmental models are widely studied, researchers occasionally discover unexpected behaviors. For instance, the standard Hodgkin–Huxley formulation might not fully explain every aspect of spike generation, and even slight additions or noise terms can lead to new details about spiking reliability or variability (Fang et al., 2021). They also cannot model dendritic computations like synaptic integration along branching processes, which is still a limitation of their speed and simplicity (Hendrickson et al., 2010; Brette, 2015).

#### 6.1.5 Cascade models (group 4)

Also known as Level IV models in some classifications or neural encoding models for sensory information, cascade models focus on the conceptual side of neural encoding rather than the biophysical mechanisms inside single neurons. They treat sensory processing and other neural computations as a sequence of mathematical operations—often linear filters, nonlinear transformations, and stochastic processes—that transform incoming signals into meaningful output. This stepwise approach is commonly applied to sensory systems (e.g., vision, audition) and is especially valuable for interpreting how neurons handle high-dimensional inputs in a feed-forward manner (Latimer et al., 2019; Meyer et al., 2017).

##### 6.1.5.1 Examples

- Linear cascade models

Often involve simple convolution or filtering steps to represent basic visual or auditory pathways.

- Nonlinear cascade models

Extend linear versions by adding adaptive or more complex transformations, allowing for phenomena like contrast gain control or adaptive coding.

- Linear–Nonlinear–Poisson (LNP) models

Include a spike generation process (Poisson) after a linear filter and a static nonlinearity, modeling how neural firing might depend on filtered stimuli (Zoltowski and Pillow, 2018; Neri, 2015).

- Generalized linear models (GLMs)

Provide a flexible framework to fit input–output relationships from empirical data, incorporating spike history effects or refractoriness.

- Hierarchical Max-Pooling models

Stack multiple filtering and pooling layers, modeling advanced visual processes (e.g., complex cells in the cortex).

##### 6.1.5.2 Key features of this group

- Conceptual level of computation
  Emphasize the functional transformations neurons perform on inputs, rather than morphological details or ion channel distributions (Koch, 1998).
- Mathematical primitives
  Rely on operations like convolution (linear filters), rectification (nonlinear functions), and random processes (e.g., Poisson spiking) (Moskovitz et al., 2018). For instance, adding a normalization nonlinearity to cascaded linear filters can capture motion processing in visual pathways, illustrating how these transformations go beyond simple linear filtering (Simoncelli and Heeger, 1998).
- Fitting to experimental data
  Model parameters are often obtained through regression or maximum likelihood methods (Pillow et al., 2008).
- Applications beyond sensory pathways
  Although widely used in vision or audition, they can also address how neurons adapt to different stimulus statistics or encode multiple features (Betzel et al., 2024).

##### 6.1.5.3 Potential applications for brain implants

1. Sensory restoration
  Translating sensory signals through cascaded filtering and nonlinearity may let devices (e.g., cochlear or retinal implants) approximate natural coding (Zrenner, 2002; Wilson and Dorman, 2008; Fornos et al., 2019). However, real neural circuits include feedback and context-dependent processing that simple cascades do not capture.
2. Signal processing chains
  Each stage can be optimized independently, which might simplify the design of implant firmware that manages noisy or high-dimensional signals. But strongly sequential structures may not adapt well to dynamic conditions (and neuromorphic parallel nature) involving recurrent loops or feedback from other brain areas (Guo et al., 2024; Lebedev and Nicolelis, 2017).
3. Adaptive tuning
  Modularity of these models makes it easier to adjust individual layers to reflect patient-specific changes in neural responses. However, if the implant requires fully online adaptation, the computational overhead of re-fitting multiple parameters might be too high for hardware with strict power constraints (Dehghanzadeh et al., 2021; Fisher et al., 2023).
4. Conceptual simplicity
  Cascade-based frameworks can run efficiently on neuromorphic chips in feed-forward mode, aligning with the power constraints typical of implantable devices (Dehghanzadeh et al., 2021). Yet they might not handle recurrent or complex feedback-driven behaviors (e.g., certain cognitive tasks) within the same model architecture (Guo et al., 2024).

##### 6.1.5.4 Limitations

Cascade models primarily handle forward-flow transformations, which may be insufficient for neurons deep in sensory pathways or for tasks that involve complex feedback loops. While they are well-suited for discovering or modeling receptive fields and filter stages, they might generalize poorly across diverse stimulus conditions or dynamic contexts. Additional layers (e.g., recurrent or adaptive modules) or advanced model structures may be necessary to handle feedback mechanisms or strong interactions among distant neural populations (Almasi et al., 2022; Zhang et al., 2017).

#### 6.1.6 Black box models (group 5)

Black box models concentrate on system-level input–output relationships, often through probability distributions such as p(Response∣Stimulus). Rather than simulating a neuron’s internal biophysical processes, these frameworks focus on functional accuracy—how well the output matches observed data or desired performance (Saxena et al., 2012; Sheu, 2020). Because they do not require detailed morphological or mechanistic information, they can adapt to various conditions by learning directly from empirical data (Saadatinia and Salimi-Badr, 2024).

##### 6.1.6.1 Examples

- Input–output models
  Characterize stimulus–response mappings from recorded data, with no aim to explain the neuron’s internal workings.
- Neural network models
  Aim to maximize performance on tasks like classification or regression, sometimes using large training datasets.
- Support vector machines (SVMs)
  Use margin-based optimization to separate classes or predict continuous outputs, staying agnostic about neuronal details.
- Gaussian process models
  Provide a probabilistic treatment of inputs and outputs, offering uncertainty estimates and flexible nonparametric fitting.

##### 6.1.6.2 Key features of this group

- Neglect of biophysical mechanisms
  They intentionally bypass the ion channels, compartments, or morphological aspects of neurons, focusing solely on mapping from inputs to outputs (Karim et al., 2023).
- Data-driven probability distributions
  Often define the relationship p(R∣S) between response (R) and stimulus (S), sometimes relying on nonparametric methods that infer distributions directly from measurements (Huang, 2024).
- Adaptability & neural efficiency
  Useful for examining how operating points shift when input statistics change, or how the system evolves to maintain robust performance (Karim et al., 2023).
- Functional outcomes
  Emphasize results like error rates, decoding accuracy, or predictive power over explanations of *how* these results emerge biologically. However this is shifting in recent years toward more explainable models.

##### 6.1.6.3 Applications in brain implants

1. Functional goals
  When the primary objective is to achieve a specific outcome—for example, schizophrenia diagnosis—black box methods can deliver good performance without detailing internal neuronal mechanisms (Saadatinia and Salimi-Badr, 2024; Highton et al., 2024). However, since these models do not rely on biological details, they may struggle to provide precise or fine-tuned stimulation patterns that align with actual neural processes.
2. Rapid design and high-level control
  Quick to train or configure, making them a practical choice for developing algorithms for prosthetics or brain–machine interfaces with minimal assumptions about the neuron-by-neuron architecture (Lebedev and Nicolelis, 2017). But it may require frequent retraining when faced with varied stimuli or changing patient conditions, which can be demanding on implant hardware making it more practical for software use so not on chip computing.

##### 6.1.6.4 Limitations

Neuromorphic hardware often restricts how plasticity is implemented, limiting real-time learning for black box models. Real-time performance in a changing physiological environment demands adaptive algorithms, which can be difficult to support on-chip (Mayr et al., 2016). In practice, it could be more feasible to train networks offline, then deploy fixed weights on custom neuromorphic chips, achieving efficient inference but reducing in-situ adaptability (Merk et al., 2023).

#### 6.1.7 Unifying models: fundamental code unit and brain code

The Fundamental Code Unit (FCU) and Brain Code (BC) frameworks provide a method for connecting low-level biological processes (e.g., ion channels, protein interactions) with high-level cognitive outputs (e.g., language, decision-making). They link detailed and abstract perspectives, describing how neural signals move from molecular chirality in neurotransmitters to fully formed mental activities in human cognition (Howard and Hussain, 2018; Howard et al., 2020; Isik et al., 2024).

##### 6.1.7.1 Key features

1. Higher-Order Abstractions
  - FCU is presented as an abstract code unit that relates basic biophysical events (like spikes or protein-driven signaling) to more advanced mental functions. This approach covers multiple scales, from neurochemical to behavioral.
2. Four Principles of Brain Code
  a) Activation Thresholds: Includes phenomena such as action potential thresholds, Weber’s just-noticeable differences, and baseline neural firing cutoffs.
  b) Duration of the Signal: Considers how long a signal persists, influencing short-term loops and long-term patterns.
  c) Waveforms for Information Transfer: Addresses oscillatory or spike-based signals, including the influence of neurotransmitter chirality.
  d) Transduction Between Different Forms of Energy: Focuses on transitions such as ATP/ADP cycles and how chemical gradients become electrical impulses.
3. Brownian Motion and Protein Dynamics
  a) Recognizes the stochastic nature of neurotransmission—e.g., in the ubiquitin–proteasome or autophagy–lysosome pathways—and how it affects signal consistency.
4. Cumulative Cognitive Output
  a) Treats language and behavior as products of ongoing neural signals, showing how short-term electrical events connect to higher cognitive or emotional states (e.g., isomer-specific mood shifts).
5. Stochastic Neural Signals
  a) Notes that spikes, channel gating, and synaptic release have random components, and that the ON/OFF unary math in FCU can incorporate these variations.
6. Relation to Memory and Learning
  a) Connects with processes such as LTP (long-term potentiation), LTD (long-term depression), and ATP-related energy usage to account for extended neural plasticity in cognition.
Unary Mathematics

- Uses a ± coding approach to simplify neural signaling into an ON/OFF framework, designed for efficient implementation in computational hardware.

##### 6.1.7.2 Applications for brain implant chips

1. Better signal decoding

By converting noisy or random neural activity into a unary code, implants may decode signals with improved consistency while staying within practical hardware limits.

2. Improved treatment of neurological disorders

Tuning stimulation parameters based on details like molecular chirality or activation thresholds could help devices manage conditions (e.g., Parkinson’s), adjusting activity patterns as the brain state evolves (Howard and Hussain, 2018).

3. Predictive modeling

Incorporating Brownian motion and stochastic synaptic events might allow early detection of cognitive or pathological shifts, enabling preemptive intervention.

4. Integrated data processing

Combining sensor inputs, linguistic data, and behavioral metrics within a unary-coded structure offers a more unified view of a patient’s neural and cognitive status (Howard and Hussain, 2018).

##### 6.1.7.3 Limitations

Some limitations might include that parameters rely on *in vitro* data, raising questions about how well they generalize to *in vivo* conditions. Moreover the models need robust methods for extracting relevant neural features and translating them to control mechanisms.

#### 6.1.8 Combining models

While each modeling level has its own strengths—from the spatial detail of Detailed Compartmental Models to the high-level functionality of Black Box approaches—their components could possibly be unified on a single neuromorphic chip to address a plethora of brain implant needs, considering the chip’s power restrictions (Dehghanzadeh et al., 2021; Qi et al., 2023). For real-time loops (e.g., motor control or seizure detection), Single-Compartment Models or Reduced Compartmental Models may handle essential dynamics under strict power and latency constraints, while Cascade Models can structure sensory signal flow in specialized stages (Ramezanian-Panahi et al., 2022). Meanwhile, Fundamental Code Unit (FCU) and Brain Code (BC) frameworks can integrate higher-level or axiological factors for adaptive therapies (e.g., shifting stimulation patterns in response to cognitive changes) (Howard and Hussain, 2018). Where long-term plasticity is required, custom or STDP-based rules could let the system change stimulation over time as a patient’s neural state changes. A combination of parameters from various models is necessary for building effective on-chip computing for the brain implant (Bazzari and Parri, 2019).

One key challenge for brain implants is scaling from micro-level neuronal activity to macro-level outcomes—such as conscious arousal or sleep states—an aspect already explored by FCU and BC. Recent studies show how single-neuron biophysics and large-scale arousal states connect, demonstrating how microscopic spiking or bursting patterns can influence macroscale phenomena like wakefulness or anesthesia (Munn et al., 2023; McGinley et al., 2015). This highlights the need to design computational systems that merge compartmental or spiking neuron models with higher-level frameworks in neuromorphic designs, particularly for brain implants intended for adaptive real-time control across different brain states. In this way, researchers move beyond local neuronal or therapeutic effects to consider how large-scale behaviors might be altered—and the possible risks involved—with more research being conducted (Munn et al., 2023; Singh et al., 2025; Zotey et al., 2023). An alternative approach could be modeling the state of neural disease with a gradual progression toward a healthy model, allowing for controlled healing of neural matter.

Malfunction of these higher-level models (the formal information-processing rules) or the underlying mechanisms (the neural signal transformations themselves) can similarly impact executive cognition, as recent study shows (Barack and Platt, 2016). When models fail to execute correctly, or when neural circuits (e.g., in medial prefrontal cortex) malfunction and misimplement those computations, the result may be dysfunctional behavior or impaired cognition. For brain implants seeking to restore or enhance higher-order functions, it is important to consider not just how a desired model is defined, but also how the actual neuronal mechanisms implement it in real time.

### 6.2 Challenges of neurocomputational models on neuromorphic hardware

#### 6.2.1 Complexity of neural dynamics simulation

Many neurocomputational models—particularly those simulating detailed neural activity like ion channels or synaptic integration—require significant computational resources (Hodgkin and Huxley, 1952; Koch, 1998). Current neuromorphic hardware might lack the complexity or precision to manage these dynamics at scale, forcing simplifications that reduce fidelity (Schuman et al., 2017; Ward and Rhodes, 2022). This is especially problematic for brain implants, where accurate neural modeling is crucial for addressing pathologies. Different neuron types are determined by gene expression, their specific ion-channel complement, and the polarity of the intra- and extracellular environment connecting all parts of the cell. Ion channels can exhibit diverse timescales, voltage ranges, or states of inactivity, shaping how neurons respond to inputs and external modulation (Koch, 1998).

Designing neuromorphic interfaces that generate adaptable and safe electrical patterns is also challenging. While the brain can adapt to varied signals, correctly reproducing individual cell dynamics and modeling the bioelectrical environment might help lower the risk of immune responses (Lebedev and Nicolelis, 2006; Polikov et al., 2005; Fani et al., 2023). These implants must deliver stimuli with precisely defined amplitude and timing, adjusting to ongoing brain activity. Achieving this depends on understanding how different neural pathways contribute to sensory processing, cognition, and action, then using real-time algorithms to interpret signals and convert them into suitable stimulation protocols (Contreras et al., 2024; Chiappalone et al., 2022).

#### 6.2.2 Large-scale network architectures

The complexity of brain modeling remains high, and our current grasp of neural systems continues to evolve—especially regarding regeneration, adaptation, and large-scale network modeling. Simplified models risk omitting key aspects of neural dynamics, reducing an implant’s adaptability over time as neurobiological knowledge advances (Pathak et al., 2022). Meanwhile, current neuromorphic chips face practical limits on the number of neurons and synapses they can handle due to chip size, power usage, and interconnectivity challenges (Dehghanzadeh et al., 2021; Contreras et al., 2024). Modeling entire cortical areas may require scaling down model complexity or distributing workloads across multiple chips, which introduces latency and synchronization hurdles that can constrain real-time processing in implants (Stanslaski et al., 2012).

#### 6.2.3 Scalability and power efficiency

Perhaps the most critical area of focus is developing neuromorphic chips that remain power-efficient while supporting high-complexity neural computations. This challenge is especially pressing in brain implants that demand battery-free solutions given limited battery life and the shortcomings of wireless power transfer in extending operational longevity (Dehghanzadeh et al., 2021; Miziev et al., 2024). This issue is heightened by the fact that biological brains exhibit great efficiency that neuromorphic systems frequently fail to match at scale, leading to a trade-off between model complexity and battery life in implantable devices if on-device computing must run long-term without frequent recharging. This also raises concerns about long-term stability to maintain steady chip performance and avoid degradation, particularly for patients living with the implant for many years (Dehghanzadeh et al., 2021; Miziev et al., 2024).

#### 6.2.4 Translation of models to hardware and new hardware design

A gap persists between theoretical neurocomputational models and practical neuromorphic chip implementations. Each model’s requirements for precision, timing, and connectivity may not align with architectures typically optimized for generalized SNN operations. Researchers are now exploring designs that more directly mirror biological neural systems—potentially using additional analog components for finer synaptic control, memory technologies that reflect biological processes (Zhang et al., 2020), or even xenobots (Kriegman et al., 2020), anthrobots, robots healing damaged tissues (Hutson, 2023), or other bio-hybrid solutions (Boulingre et al., 2023; Rochford et al., 2019) as technology advances. Some are also investigating mind-simulation/uploading, where a patient’s brain state could be modeled online, possibly modeling regeneration progression and adapting implants accordingly, such as in epilepsy (Jirsa et al., 2016; Watanabe, 2023). In many cases, custom-made chip designs—possibly unique to each patient (Singh et al., 2025)—might prove necessary, making off-the-shelf solutions insufficient and pushing up development costs for brain implants.

#### 6.2.5 Need for a new complete neuromorphic pipeline

**Figure 2:**
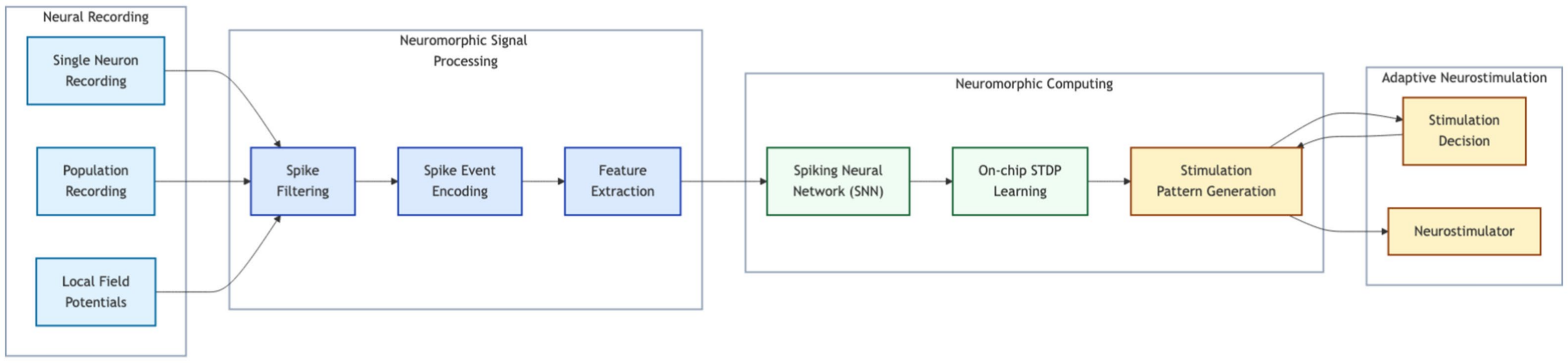
An example of a neuromorphic pipeline for brain implants, starting with neural recording and progressing through event-driven processing, spiking neural network computation, and adaptive neuromodulation for closed-loop control.

Developing a complete sensor–processor–stimulator pipeline is key for neuromorphic brain implants, as shown in Figure 2. Each component must be built specifically for neural use with possibly biocompatible materials and signal processing (Valle et al., 2024): the sensor interprets neural signals, the processor decodes and processes them in real time, and the stimulator generates precise patterns to modulate neural circuits based on the processed information (Stanslaski et al., 2012; Chiappalone et al., 2022). Neuromorphic bio-signal interfaces enhance this pipeline by enabling efficient EEG and ECG processing for real-time applications, such as decoding brain states or monitoring cardiac activity, though integrating these capabilities into a compact, low-power system is still a significant challenge (Bauer et al., 2019; Sharifshazileh et al., 2021; Li et al., 2024a,b).

## 7 Conclusion and next-generation brain implants

Addressing these issues requires the development of high-performance, low-power neural signal processing algorithms with adequate compression capabilities, though this often involves navigating trade-offs between chip size and functionality.

### 7.1 Memory storage

Memory storage in neuromorphic hardware may someday replicate the brain’s own mechanisms for encoding, storing, and retrieving information, possibly through advanced materials, such as neuromorphic optical data storage enabled by nanophotonics (Lamon et al., 2024; Tait et al., 2019), FLASH memory, other NVM technologies (Mehonic et al., 2024), or adaptable wire Creating truly effective neuromorphic systems requires a “brain code” that captures key biochemical and electrical dynamics while adapting to neural activity and plasticity (Valle et al., 2024; Chiappalone et al., 2022). Research on cellular intelligence suggests this code must surpass traditional neural models, incorporating bioelectrical signals that act as “software” guiding cellular behavior and even large-scale anatomical outcomes such as organ formation (Levin, 2021). Such competencies and collective goals create a multi-scale architecture that implants must interpret and modulate. Yet even advanced neuromorphic technologies only partially capture these emergent properties—self-organization and goal-directed behavior. Success will hinge on fidelity and adaptability: model choices must respect synaptic plasticity, network connectivity, and biochemical responsiveness without disrupting the brain’s natural processes (Contreras et al., 2024; Rommelfanger et al., 2021).

### 7.2 Single spatial resolution and adverse effect

Implementing neuron-to-neuron modeling rather than stimulating a broader area may be important for achieving better therapeutic outcomes (Zhang et al., 2023; Topalovic et al., 2023; Campbell et al., 2024). ETH Zurich researchers have found that neuroprosthetics work better when using signals inspired by nature, proving superior to time-constant stimulation in the case of leg amputees while being less demanding for the brain (Valle et al., 2024).

Although it may seem conceivable that the brain could adapt to any kind of electric signal or pattern, cells react to mechanical properties in their surroundings—including matrix stiffness and external forces—and targeting entire clusters instead of a single dysfunctional cell can trigger unwanted reactions (Kumosa, 2023; Su et al., 2024; Davidson et al., 2021; Ladoux and Mège, 2017; Alberts et al., 2002). Broad stimulation risks overlooking the collective intelligence of cellular networks, where cells communicate via bioelectrical and molecular signals to achieve specific goals (Levin, 2021). Disrupting this collective behavior could amplify adverse effects by ignoring the emergent dynamics that govern tissue integrity and function. Interconnected cells influence each other’s behavior, including through extracellular and systemic signaling. As a result, stimulating an entire group can amplify the release of harmful molecules from dysfunctional cells—compromising tissue integrity—or trigger systemic immune responses, potentially leading to inflammation or hypersensitivity disorders. It may also cause therapeutic effects to lose selectivity or become counterproductive if normal cells are affected, but more research is required to further confirm this (Su et al., 2024; Hegade and Rashid, 2024; Kubelt et al., 2021; Alberts et al., 2002). Moreover, the potential impact of the cerebral vascular network when designing and implanting devices should be taken into account—not only during the implant’s design but also as a potential source of noise or fluctuation in signal recording (Kozai et al., 2014).

1. Neighboring cell effects
  Neighboring cells often evaluate each other’s fitness through mechanisms like “fitness fingerprints,” where less fit cells are targeted for elimination by their healthier neighbors. Stimulating all cells in a group could disrupt this balance and potentially enhance the survival of pathological cells or suppress the natural elimination of damaged ones (Madan et al., 2019; Colom et al., 2020; Rao et al., 2012).
2. Release of toxic substances
  When dysfunctional cells are stimulated, they may release harmful substances such as reactive oxygen species or inflammatory signals, which can damage surrounding normal cells. For example, dying neurons release neurotoxic factors that harm nearby neurons, and preventing this through targeted interventions can potentially protect the group (Rao et al., 2012; Lee et al., 2010; Block and Hong, 2005).
3. Immune system activation
  Broad stimulation may inadvertently activate immune responses, such as T-cell hypersensitivity reactions. These reactions can lead to systemic effects like inflammation or tissue damage, as seen in drug-induced hypersensitivity reactions mediated by off-target immune receptor interactions (Pichler et al., 2015; Adam et al., 2010; Wuillemin et al., 2022).
4. Off-target effects
  Stimulating a group of cells could result in unintended activation of nearby normal cells, leading to off-target effects. For instance, with the simultaneous intake of drugs, interactions with non-target receptors or proteins can cause unpredictable side effects, including immune-mediated adverse reactions (Adam et al., 2010; Wuillemin et al., 2022).
  Future advancements must decode cellular communication—understanding collective goals and incorporating multi-scale, non-invasive approaches to achieve full integration with the brain’s complex intelligence and architecture. This shift, potentially through biohybrid solutions or enhanced neuromorphic models, requires further studies to bridge the gap between current technology and the brain’s capabilities. More research needs to be done to study the effects of targeted neuromorphic stimulation and potential adversarial effects of large surface stimulation, particularly in light of cellular communication and collective biological intelligence for next-generation implants.
